# Laparoscopic surgery for appendiceal cancer with intestinal malrotation in an adult: A case report

**DOI:** 10.1016/j.ijscr.2020.12.068

**Published:** 2020-12-24

**Authors:** Hirokatsu Hayashi, Mamoru Matsuhisa, Yusuke Murase, Hitoya Sano, Kimitosi Nishio, Iwao Kumazawa

**Affiliations:** Department of Surgery, JA GIFU Kouseiren Ibi Kosei Hospital, 2547-4 Miwa, Ibigawa-cho, Ibi-district, Gifu-Prefecture, 501-0696, Japan

**Keywords:** 3D, three-dimensional, CT, computed tomography, SMA, superior mesenteric artery, Appendiceal cancer, Intestinal malrotation, Laparoscopic surgery

## Abstract

**Introduction:**

Intestinal malrotation is a congenital anatomical anomaly resulting from abnormal midgut rotation. Many cases occur during childhood and present with intestinal obstruction and midgut volvulus. Intestinal malrotation rarely occurs in adults and is found incidentally because it is asymptomatic. We herein report a case of intestinal malrotation, and colorectal cancer operated laparoscopically.

**Presentation of case:**

A 78-year-old man presented to our Department of Surgical Gastroenterology with fecal occult blood. There were no abnormal findings in the physical examination. Colonoscopy revealed a type 3 tumor in the cecum. Contrast-enhanced computed tomography revealed that the tumor was located in the appendix along the midline of the abdomen. The small intestine and colon occupied the right and left sides of the abdominal cavity, respectively. The diagnosis was appendiceal cancer with nonrotation-type intestinal malrotation. A laparoscopy-assisted ileocecal resection was performed. During surgery, the right-side colon was not fixed to the retroperitoneum, and the right-side colon could be extracted out of the abdominal cavity through the umbilical wound with only adhesive dissection, and mesenteric and lymph node dissection can be performed outside the body. The postoperative course was uneventful.

**Discussion:**

Appendiceal cancer with intestinal malrotation is managed with laparoscopic surgery because this method is safe and minimally invasive.

**Conclusion:**

The laparoscopic approach may be safer and less invasive than laparotomy, and extracorporeal lymph node dissection is safe and reliable for patients with intestinal malrotation.

## Highlights

• Laparoscopic surgery may be safer and less invasive than laparotomy.
• Understanding anatomical abnormalities is useful to plan lymph node dissection.
• Extracorporeal lymph node dissection is useful in cases of intestinal malrotation.

## 1 Introduction

Intestinal malrotation is a congenital anatomical anomaly resulting from abnormal rotation of the midgut. It may often present in childhood with intestinal obstruction and midgut volvulus [1]. In adults, intestinal malrotation is rarely present and is found incidentally at the time of gastrointestinal examination or operation because it is asymptomatic [2].

We herein report a patient with intestinal malrotation and colorectal cancer who had undergone laparoscopic surgery. This work has been reported in line with the SCARE criteria [3].

## 2 Presentation of case

**Fig. 1:**
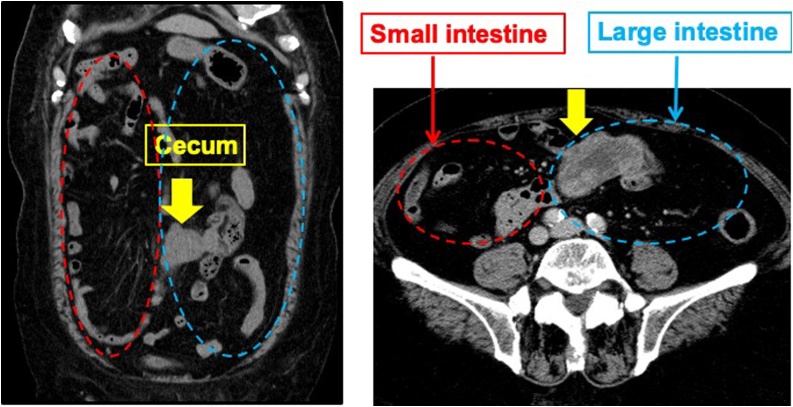
Contrast-enhanced CT. The images show the thickness of the appendiceal wall, which is located along the midline of the abdomen. The small intestine and colon are at the right and left sides of the abdominal cavity, respectively.

**Fig. 2:**
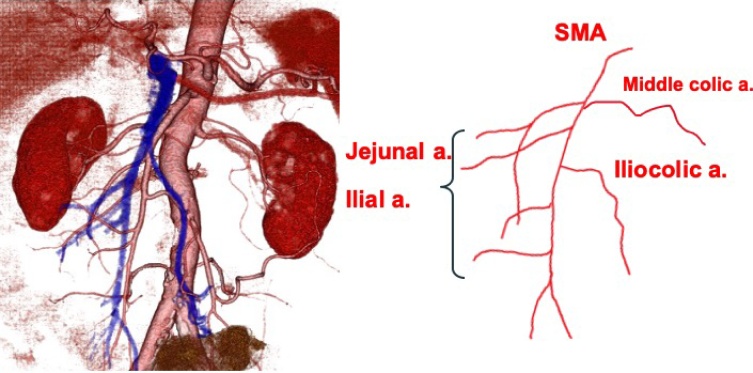
3D-CT angiography. The jejunal and ileal arteries and ileocolic and middle colic arteries branch from the right and left sides of the SMA, respectively.

**Fig. 3:**
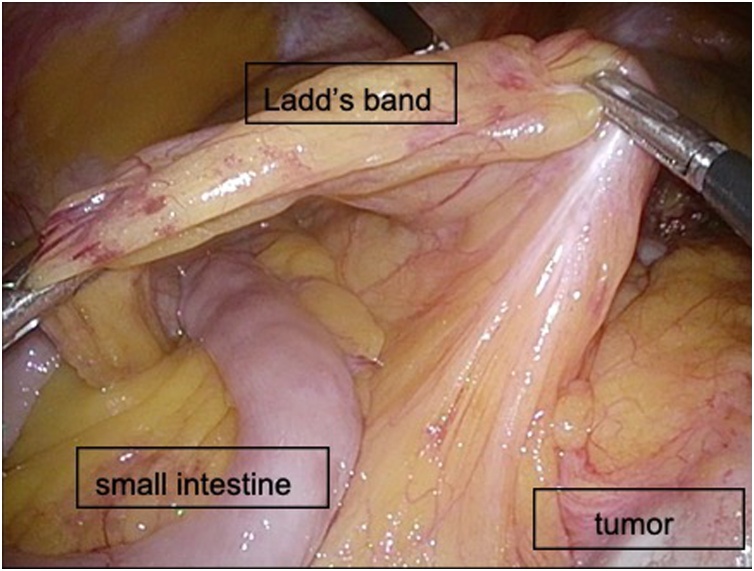
Ladd’s band in front of the small intestine. Ileocecal region is located along the midline of the abdomen.

**Fig. 4:**
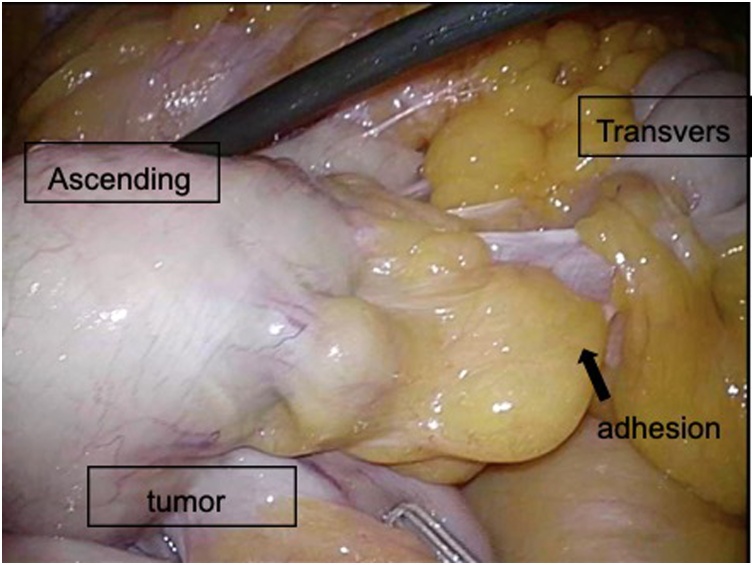
The ascending colon has adhesions with the greater omentum, transverse colon, and Ladd’s band.

**Fig. 5:**
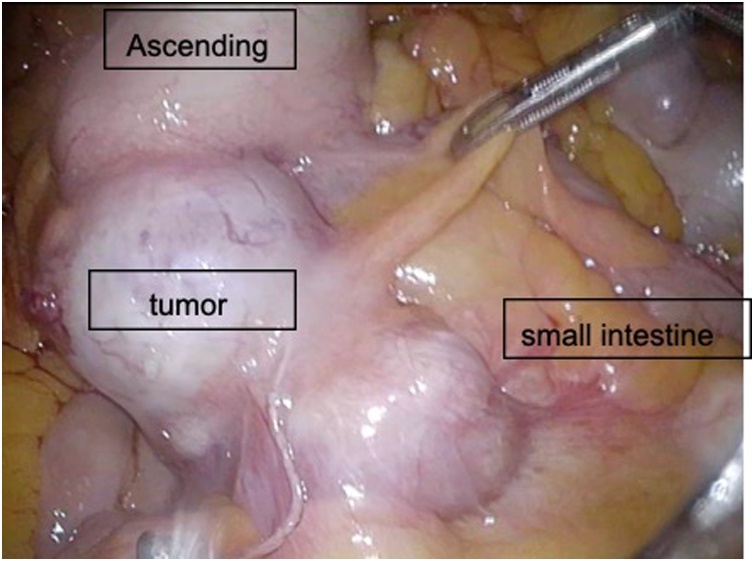
The appendiceal cancer is located along the midline of the abdomen.

**Fig. 6:**
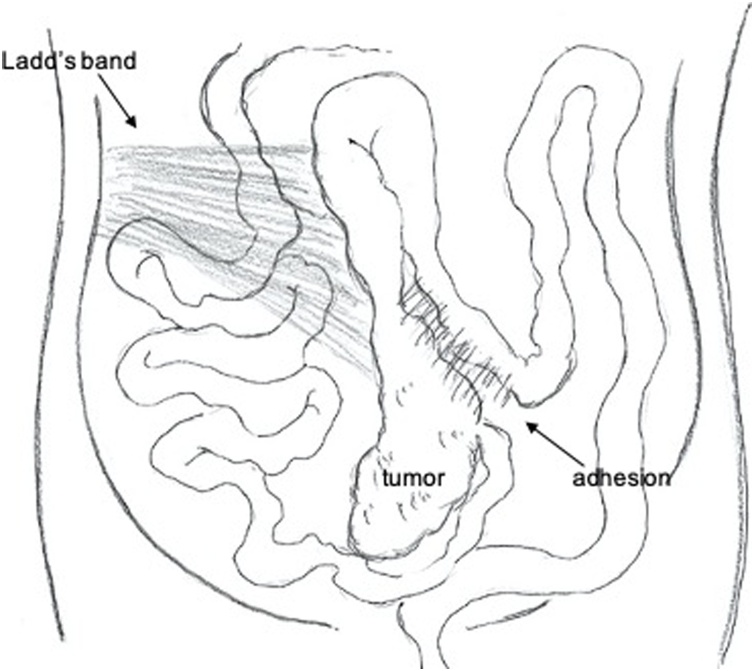
Depiction of intraoperative findings of intestinal malrotation.

A 78-year-old man presented to our Department of Surgical Gastroenterology, unassisted, with fecal occult blood. He had a past medical history of hypertension and diabetes mellitus, having been prescribed antihypertensive and antidiabetic medicines, and there was no relevant family history. He has never had any abdominal related surgery before. There were no abnormal findings in the physical examination. Colonoscopy revealed a type 3 tumor in the cecum, which was confirmed as an adenocarcinoma. His serum carcinoembryonic antigen level was 3.5 ng/mL, and his carbohydrate antigen 19-9 level was 4.6 ng/mL. Other laboratory data showed no abnormalities. Contrast-enhanced computed tomography (CT) revealed the thickness of the appendiceal wall, which was located along the midline of the abdomen, without lymph node swelling or metastatic lesions. The small intestine and colon occupied the right and left sides of the abdominal cavity, respectively (Fig. 1). Three-dimensional (3D)-CT angiography showed that the jejunal and ileal arteries, and ileocolic and middle colic arteries branched from the right and left sides of the superior mesenteric artery (SMA), respectively (Fig. 2). The diagnosis was appendiceal cancer with non-rotation-type intestinal malrotation. We scheduled a laparoscopy-assisted ileocecal resection. Intraoperative examination revealed that the third and fourth parts of the duodenum descended vertically without the ligament of Treitz, and the small intestine was located on the right-side of the abdominal cavity. The ascending colon and cecum were not fixed with the retroperitoneum and were located along the midline of the abdomen. The ascending colon had adhesions with the greater omentum, transverse colon, and Ladd’s band (Figs. 3, 4, 5 and 6). After adhesive dissection, the ileocecal region was extracted out of the abdominal cavity through the umbilical wound and ileocecal resection with D2 lymph node dissection was performed outside the body. Histopathological examination revealed a mucinous adenocarcinoma of the appendix penetrating the muscularis propria with lymphatic and vascular invasion and metastatic involvement in 1 of the 6 dissected lymph nodes. According to the tumor-node-metastasis classification of malignant tumors, the diagnosis was Stage IIIa (T3N1aM0). Capecitabine (3000 mg/day) was administered as adjuvant chemotherapy, which was completed in only one course due to deterioration of renal function. The patient is still being followed up at our hospital, with no recurrence or distant metastases observed using CT or blood tests at 18 months after surgery.

## 3 Discussion

Intestinal malrotation is a congenital abnormal rotation in the midgut. During embryologic development, the midgut rotates 270° counterclockwise around the SMA and fixes to the retroperitoneum [4]. Depending on the degree of rotation, intestinal malrotation is classified into four types: nonrotation (90° counterclockwise rotation), malrotation (180° counterclockwise rotation), reversed rotation (90° clockwise rotation), and paraduodenal hernia [5].

Intestinal malrotation is found in 0.01–0.02% of infants, and 80% of cases present with midgut volvulus or intestinal obstruction in the first few weeks of life [1]. In adults, most cases of intestinal malrotation, of which the nonrotation-type is the most frequent, are incidentally found at the time of digestive tract examination or operation because it remains asymptomatic [2,6].

Intestinal malrotation diagnosed in adulthood is rare, and the recent method of diagnosis is CT [7]. Intestinal malrotation is associated with specific radiological findings. The duodenum has a vertical path that does not cross the midline, and the small intestine is located on the right side of the abdominal cavity. The colon is located on the left-side of the abdominal cavity, and vessels of the right-side colon bifurcate from the left-side of the superior mesenteric vessel. The superior mesenteric vein (SMV) is located on the left-side of the SMA, which is termed the SMV rotation sign [8].

Reports have indicated that in complications of right-side colon cancer, chronic intestinal obstruction caused by anatomical disorders of the colon leads to inflammation and carcinogenesis [9]. Literature search revealed intestinal malrotation with colon cancer in 55 cases from 1974 to 2018 in Japan, and only 11 other cases have been reported worldwide [[10], [11], [12], [13]]. A total of 37 cases of right-side colon cancer, including the appendix, cecum, ascending colon, and transverse colon, have been reported. A total of 19 cases of left-side colon cancer, including the descending colon, sigmoid colon, and rectum, have been reported.

With respect to the surgical approach for intestinal malrotation with right-sided colon cancer, 31 cases of laparotomy and 16 cases of laparoscopic surgery have been performed (Table 1). In the last 10 years, the use of laparoscopic surgery has been increasing. The advantages of laparoscopic surgery include its ability to provide information about the entire abdominal cavity. This allows a variety of approaches to be performed without the need for a large skin incision. However, at the time of surgery, anatomical abnormalities of vessels and adhesions are problematic. 3D-CT angiography is a useful modality to understand anatomical abnormalities of vessels and to plan reliable lymph node dissection. Adhesions between the intestinal tracts and peripheral organizations are often present and require dissection. However, in many cases, the fixation of the right-side colon to the retroperitoneum is weak. Thus, the colon can be extracted out of the abdominal cavity through the umbilical wound with only adhesive dissection, and mesenteric and lymph node dissection can be performed outside the body. In fact, extracorporeal dissection was performed in 9 cases.

**Table 1:** Review of literature of intestinal malrotation with right-side colon cancer treated by laparoscopic surgery.

	Author	Year	Location	Type	Malrotation diagnosed	Staging	Mesenteric excision	Lymph node dissection
1	Yamamoto	2007	Ascending colon	nonrotation	CT scan, enema	MP,N0,M0,StageI	outside body	D3
2	Takahashi	2009	Ascending colon	nonrotation	CT scan	SS,N0,M0,StageIIA	inside body	D2
3	Tokai	2012	Transverse colon	nonrotation	CT scan, enema	M,N0,M0,Stage0	outside body	D2
4	Nakatani	2013	Cecum	nonrotation	operation	SS,N0,M0,StageIIA	outside body	D1
5	Hirano	2013	Transverse colon	reversed rotation	CT scan, enema	SM,N0,M0,StageI	outside body	D2
6	Hirano	2013	Ascending colon	malrotation	CT scan, enema	M,N0,M0,Stage0	outside body	D2
7	Sakaguchi	2013	Cecum	nonrotation	operation	SS,N0,M0,StageII	inside body	D1
8	Takahashi	2014	Ascending colon	malrotation	CT scan, enema	SS,N1,M0,StageIIIa	outside body	D3
9	Enomoto	2104	Transverse colon	nonrotation	CT scan	SS,N0,M0,StageIIa	inside body	D3
10	Kuroda	2014	Transverse colon	nonrotation	CT scan	SE,N1,M0,StageIIIa	inside body	D3
11	Morioka	2015	Cecum	nonrotation	CT scan	SS,N0,M0,StageII	inside body	D3
12	Kuwahara	2015	Transverse colon	nonrotation	CT scan	SS,N0,M0,StageII	inside body	D3
13	Motoki	2016	Ascending colon	nonrotation	operation	MP,N0,M0,StageI	outside body	D2
14	Nakatani	2017	Cecum	nonrotation	operation	M,N0,M0,Stage0	outside body	D1
15	Takahashi	2017	Ascending colon	malrotation	operation	SM,N0,M0,StageI	inside body	D3
16	Kiya	2017	Cecum	malrotation	CT scan	MP,N0,M0,StageI	inside body	D3
	Hayashi		Cecum	nonrotation	CT scan, enema	SS,N1,M0,StageIIIa	outside body	D2

The learning point in this case is that laparoscopic surgery should be considered as the first choice for patients with intestinal malrotation because it is minimally invasive and allows for a variety of approaches. In addition, extracorporeal mesenteric and lymph node dissection should be performed because of the ease of adhesion dissection.

## 4 Conclusion

We believe that the laparoscopic approach is safer and less invasive than laparotomy. We also believe that extracorporeal lymph node dissection is a safe and reliable method for patients with intestinal malrotation.

## Declaration of Competing Interest

The authors report no declarations of interest.

## Sources of funding

This research did not receive any specific grant from funding agencies in the public, commercial, or not-for-profit sectors.

## Ethical approval

This report was reviewed and approved by the Institutional Review Board of JA GIFU Kouseiren Ibi Kosei Hospital.

## Consent

Informed consent was obtained from the patient for publication of this case report.

## Author contribution

Hirokatsu Hayashi: Data Acquisition, Data Interpret and writing of the manuscript.

Mamoru Matsuhisa: management of case.

Yusuke Murase: management of case.

Hitoya Sano: management of case.

Kimitosi Nishio: Supervision, review and editing.

Iwao Kumazawa: Supervision, review, editing, and final approval of the version to be submitted.

## Registration of research studies

N/A.

## Guarantor

The Guarantor is Hirokatsu Hayashi.

## Provenance and peer review

Not commissioned, externally peer-reviewed.
